# NIR Spectroscopic Properties of Aqueous Acids Solution

**DOI:** 10.3390/molecules17067440

**Published:** 2012-06-15

**Authors:** Ahmad Fairuz Omar, Hanafi Atan, Mohd Zubir MatJafri

**Affiliations:** 1 School of Distance Education, Universiti Sains Malaysia, 11800 Penang, Malaysia; Email: ahanafi@usm.my; 2 School of Physics, Universiti Sains Malaysia, 11800 Penang, Malaysia; Email: mjafri@usm.my

**Keywords:** citric acid, malic acid, NIR, oxalic acid, pH, tartaric acid

## Abstract

Acid content is one of the important quality attributes in determining the maturity index of agricultural product, particularly fruits. Despite the fact that much research on the measurement of acidity in fruits through non-destructive spectroscopy analysis at NIR wavelengths between 700 to 1,000 nm has been conducted, the same response towards individual acids is not well known. This paper presents NIR spectroscopy analysis on aqueous citric, tartaric, malic and oxalic solutions through quantitative analysis by selecting a set of wavelengths that can best be used to measure the pH of the solutions. The aquaphotomics study of the acid solutions has generated R^2^ above 0.9 for the measurement of all acids. The most important wavelengths for pH are located at 918–925 nm and 990–996 nm, while at 975 nm for water.

## 1. Introduction

Acidity and soluble solids content are the common quality attributes that are associated with the maturity index of agricultural products, especially fruits. The measurement of these attributes assists in determining appropriate harvesting time, methods for postharvest handling and supply chain management. Non-destructive spectroscopy measurements of these attributes have been applied widely by researchers worldwide. Nicolai *et al*. [1] have summarised spectroscopic measurement results and analysis that have been conducted on various types of fruits and vegetables over the last two decades. The following are the outline of the advantages of wavelengths between 700 nm and 1,100 nm (the range of NIR wavelengths that have been used in this research) for quality measurements of intact fruits [2,3,4,5,6].

(1) Penetration of the radiation into sample is deeper than other ranges.
(2) Water absorbance peaks are less strong and broad than in other ranges and the risk of masking spectral information correlated to low concentration constituents is low.
(3) The cost of instrumentation for this range is relatively low, it is portable and suitable for process control and for *in situ* field measurements.
(4) The bands are ascribed to the third and forth overtones of O-H and C-H stretching modes and are expected to be separated due to anharmonicity (approximately around 700–800 nm [7,8,9]
(5) Lower absorbance at these wavelengths allows transmission optics.
(6) Moreover, there is strong evidence, that the range from 700–900 nm constitutes a “*diagnostic window*” in which chemical compositions of samples can be investigated.

For soluble solids content in fruits, the major measurand is the mixture of sugars (sucrose, glucose and fructose) and water. There are various NIR spectroscopy experiments that have been conducted for characterising water-sugar solutions such as those by Bakier [10], Giangiacomo [11], Buning-Pfaue [12], Rodriguez-Saona [13], Berentsen *et al.* [14], Rambla *et al.* [15] and Luck [16]. However, despite the fact acidity is one of the most important attributes for the agricultural industry and there are a wide range of spectroscopy experiments that have been conducted in evaluating food acidity, the NIR spectroscopy characteristics of simple water-acid solutions are not well known. For instance, Gonzalez-Caballero *et al.* [17] have conducted spectroscopy experiments for the measurement of grape acidity (pH). According to them, the NIR wavelengths with the greatest weight for acid measurement are 768 nm, which is associated with the C–H stretch fourth overtones, and 986 nm, which is associated to the second overtone of O–H in sugars. In another experiment, Shao and He [18] claimed that 910–925 nm is an important wavelength range for acidity prediction in bayberry juice. Liu *et al.* [19] have applied NIR spectroscopy to assess the pH of rice wines. From their work, 920 nm and 990–995 nm are the most suitable wavelengths for pH measurement. On the other hand, there are also several studies on the measurement of organic acids that have been conducted on fruit juices such as a research conducted by Xie *et al.* [20] where they have applied high performance liquid chromatography (HPLC) and NIR spectroscopy between 800–2,400 nm for the measurement of citric and malic acids to detect water-adulterated bayberry juice. Likewise, Li *et al.* [21] have applied standard enzymatic assays techniques and NIR spectroscopy between 1,100–2,500 nm for the measurement of citric acid in orange juices.

In this paper, the NIR spectroscopy analysis has been conducted for the measurement of pH of aqueous acid solutions. The main objective of this research is in identifying the combination of wavelengths that can best be used for the measurement of various acids that are commonly found in fruits, which for this research are citric (C_6_H_8_O_7_), tartaric (C_4_H_6_O_6_), malic (C_4_H_6_O_5_) and oxalic (C_2_H_2_O_4_) acid. Table 1 lists the common types of organic acids that are naturally present in fruits. Acids which occur in considerable quantities are shown in italics. In grape juice, for instance, the predominant organic acids are tartaric and malic acids, while succinic and citric acids are present in minor proportions. Organic acids with low molecular weight are an important group of compounds in juices for their influence on organoleptic properties such as flavour, colour and aroma and in the stability and microbiologic control of fruits beverages [22]. The changes in fruits organics acids are useful for checking their maturation processes [23].

**Table 1:** Organic acids that are naturally present in select fruits [24].

Fruits	Acids	Fruits	Acids
Apples	Malic, Citric	Limes	Citric, Malic, Tartaric, Oxalic
Apricots	Malic, Citric	Nectarine	Malic
Avocados	Tartaric	Orange Peel	Malic, Citric, Oxalic
Bananas	Malic, Citric, Tartaric	Orange	Citric, Malic, Oxalic
Blackberries	Malic, Citric, Oxalic	Passionfruit	Malic
Blueberries	Citric, Malic	Peaches	Malic, Citric
Cherries	Malic, Citric, Tartaric	Pears	Malic, Citric, Tartaric, Oxalic
Cranberries	Citric, Malic	Pineapples	Citric, Malic
Grapefruit	Citric, Tartaric, Malic, Oxalic	Plums	Malic, Tartaric, Oxalic
Grapes	Malic, Tartaric, Citric, Oxalic	Quinces	Malic
Kiwifruit	Citric	Strawberries	Citric, Malic
Lemons	Citric, Malic, Tartaric, Oxalic	Tangerine	Citric

According to Tsenkova [25] NIR spectra are highly influenced by pH and contain physical information such as contributions from scattering. NIR application in biological systems with high water contents such as fruits (above 80%) are strongly dominated by the spectral signature of water and will thus have peak absorptions at 760, 970, 1,190, 1,450 and 1,940 nm [10,12]. The behaviour of water at a molecular level can be explained through spectral evaluation of a whole biological sample or aqueous system under diverse conditions. The NIR spectrum of water (solvent) carries information about its solutes since they are very sensitive to the configuration and charges of the solvated molecules or clusters [25]. This paper will implement an aquaphotomics study, an area of research that is introduced to systematize the readily rich information related to the interaction between light at various wavelengths, especially within the NIR region, with aqueous systems. This will be done through Water Absorbance Pattern (WAP) by characterizing the spectral patterns of aqueous and biological systems that represent water absorbance bands which corresponds to the respective perturbations and the weight of each band in the regression equation due to the different pH values of acid solutions [25]. The robustness of wavelength selection in quantifying acids pH will be evaluated through the accuracy and precision of the predicted algorithm. 

## 2. Materials and Methods

**Figure 1:**
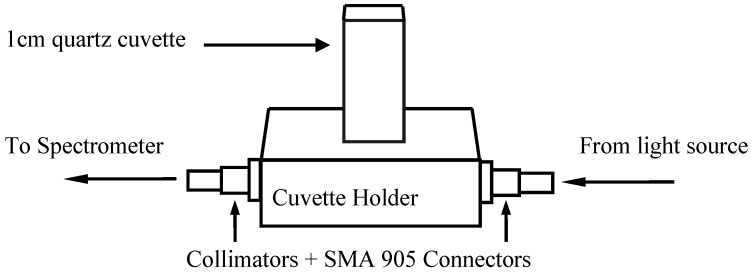
Experimental setup for absorbance measurement.

The value of absorbance was measured using a Jaz spectrometer with effective wavelength from 650 nm to 1,100 nm. Other custom setup prior to the experiment includes integration time = 3 msec, spectra averaged = 30 and boxcar smoothing = 1. Light source used was a tungsten halogen lamp with spectral emission between 360 nm and 2,000 nm and a colour temperature of 2,960 K. The reference spectrum for the absorbance measurement was collected through an empty quartz cuvette. The overall experimental setup conducted using spectroscopic instrumentations was from Ocean Optics and is illustrated in Figure 1.

The chemicals (citric, tartaric, malic and oxalic acids in powder form) was diluted using pure (reverse osmosis) water and their pH was calibrated using an ExStik pH meter (PH100) from Extech Instruments (Waltham, MA, USA) with a range of measurement between 0.00–14.00 pH, resolution of 0.01 pH and accuracy of ±0.01 pH. The pH meter was calibrated using buffer solutions with pH values of 7 and 4. The measurement unit, pH is used (and will be used through the entire research) as the measurement unit to standardise the unit of measurement for the entire research since it is one of the scientifically used units in representing acidity in fruits besides “g/100 mL” which is commonly used for titratable acidity. In this work, the response was only due to the mixtures of water with citric (Unilab Chemical), tartaric (Bendosen Laboratory Chemicals), malic and oxalic (supplied by Sinaran Saintifik Enterprise, Penang) acids. The characteristics of the acids samples used in this work are listed in Table 2. The first set of 50 acid samples was used for the development of the calibration equation. The next set of 50 samples was used to test the prediction accuracy of the developed equation. The linear relationship between optical parameters (absorbance at selected wavelengths) with the chemical composition was calculated using multiple linear regression (MLR) employing the Minitab (version 14) software to obtain the value of coefficient of determination, R^2^, where the value of R^2^ = 1 represents the theoretically perfect fit.

**Table 2:** Sample characteristics.

Acids	Formula	Range (pH)	Mean	n (calibration)	n (prediction)
Citric	C_6_H_8_O_7_	1.88–4.32	2.88	50	50
Tartaric	C_4_H_6_O_6_	2.06–4.52	3.12	50	50
Malic	C_4_H_6_O_5_	1.99–4.44	3.17	50	50
Oxalic	C_2_H_2_O_4_	1.95–4.35	3.16	50	50

## 3. Results and Analysis

**Figure 2:**
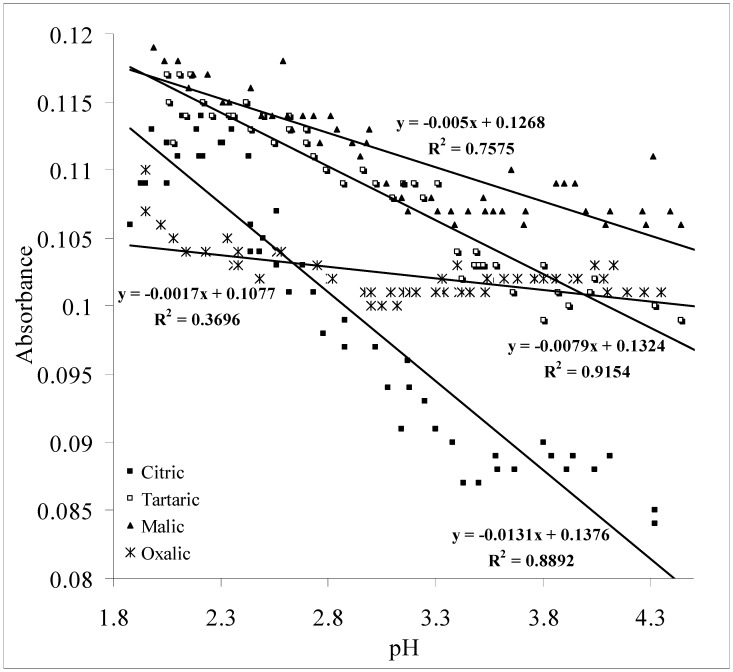
Linear relationship between absorbance and pH of aqueous citric, tartaric, malic and oxalic acid solutions at λ = 950 nm.

Light that is transmitted through an aqueous sample will attenuate in its intensity due to the absorption of light energy and also as the result of light scattered by any particles in the solution [26]. Figure 2 shows the linear relationship between absorbance and pH of aqueous citric, tartaric, malic and oxalic solutions at a wavelength of 950 nm.

One of the most critical observations from the response produced by each acid in Figure 2 is that the responsivity of pH measurement which can be represented by the slope (m) of the graph is highly correlated to the molecular size of the acids. The steepest response is produced by citric (m = 0.0131) with a single molecule that consist of 21 atoms, tartaric (m = 0.0079) 16 atoms, malic (m = 0.005) 15 atoms and oxalic (m = 0.0017) eight atoms. The “absorbance” notation in the graph does not only refer to the attenuation of light due to absorption, but also could be due to scattered light as has been stated earlier. Therefore, acid solutions with larger molecular size appear to scatter more light away from the receiving ends of the cuvette holder (to the spectrometer), thus producing a steeper response slope. Cox *et al.* [19] have conducted an experiment in determining the light scattering cross section of particles suspended in water. Particles with radii of 0.0285, 0.0605 and 0.2615 µm have been studied and the results show that particles with larger size produce higher scattering cross sections. Nonetheless, unlike the results presented by Cox *et al*. [27] where they claimed that absorption for the particles studied was negligible, in this research, the NIR attenuation could be also due to absorbance by the acid molecules. Lower pH (higher acids concentration) can be associated to higher absorption (higher loss of NIR radiation).

**Figure 3:**
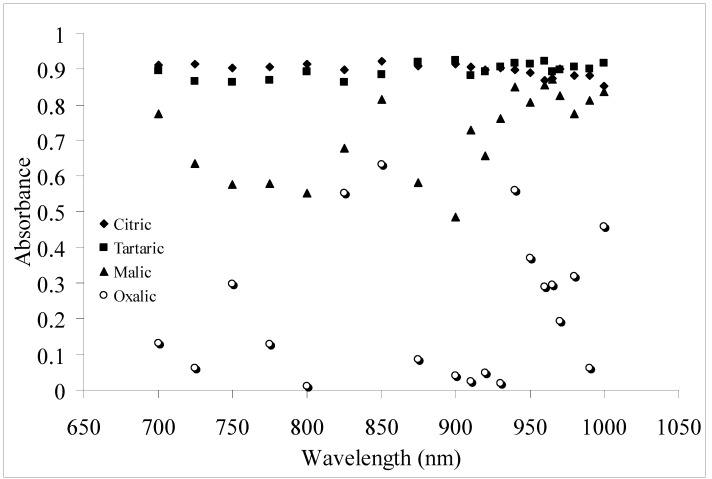
Coefficient of determination generated at different wavelengths for pH measurement of aqueous citric, tartaric, malic and oxalic acid solutions.

Technically, light at all wavelengths will be scattered when it interacts with particles. The efficiency of scattering will decay exponentially from highly scattering ability in the UV to less significant when approaching NIR. The scattered intensity is proportional to 1/λ^4^ that is based on a function of wavelength [26]. Since scattering is subjugated by physical-optical phenomenon if compared to absorption which is dominated by chemical-optical phenomena, it is inadequate for scattering effect to cause a peak response for a wavelength that lies in between non-effective wavelengths unless it is being contributed to by absorption from the molecular bonds within the sample. This is imperative in understanding and elaborating the response shown in Figure 3 where the peak response wavelength for citric acid is 850 nm (R^2^ = 0.9233), tartaric is 900 nm (R^2^ = 0.9243) and 960 nm (R^2^ = 0.9222), malic is 965 nm (R^2^ = 0.8711) and oxalic is 850 nm (R^2^ = 0.6337). For citric and tartaric, the R^2^ measured for all examined wavelengths for the measurement of pH produced high R^2^ (above 0.85) with some wavelengths producing a better result than the others. Both acids have relatively similar magnitude and shape of response. The high efficient R^2^ produced for the measurement of pH for both acids could be due to two factors:

i. Low absorptive with considerable peak at certain wavelengths while accompanied by high scattering for all wavelengths.
ii. High absorptive for all examined wavelengths with higher absorption peaks at certain wavelengths.

Malic acid produces a higher uncertainty in its response, where a peak response with R^2^ above 0.75 occurs at 700 nm, 850 nm and the rest for wavelengths between 930 nm and 1,000 nm. For oxalic acid, the highest response produced is at 850 nm with a very weak R^2^ of only 0.6337. The response produced by malic and oxalic acids show a strong indication that the responses are due to absorbance by acid molecules with no significant scattering effect.

**Figure 4:**
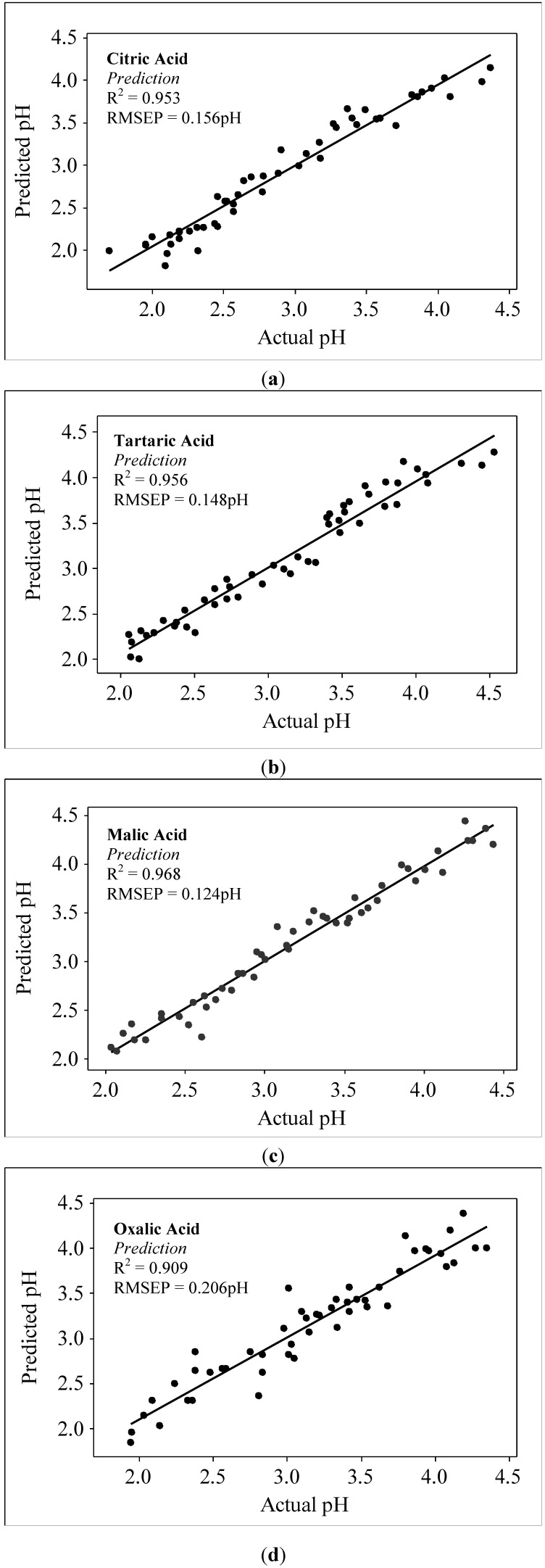
Predicted *vs.* actual pH of (**a**) Citric; (**b**) Tartaric; (**c**) Malic; (**d**) Oxalic acid.

The following discussion will dictate the combination of NIR wavelengths (between 700 nm and 1,000 nm) that can produce the best calibration algorithm in predicting the pH of aqueous acid solutions. An individual wavelength that can generate a high correlation with pH of the acid does not necessarily contribute to an increased R^2^ of the calibration algorithm when combined with other wavelengths. Therefore, the combination of wavelengths selected for the development of calibration algorithm is based on the wavelength that has a sizeable contribution in producing higher R^2^ with low RMSE. The calibration equation for all acids are represented by Equations (1)–(4) for citric, tartaric, malic and oxalic acid, respectively. The wavelength 975 nm, which is one of the peak absorptions for water, appears to be important in the development of the calibration algorithm for all types of acids examined in this research, There are two ranges of wavelengths that are seen to contribute significantly for the quantification of pH of all acids. These wavelengths are 918–925 nm and 990–996 nm. The wavelength of 850 nm is observed to contribute for citric and oxalic solutions. Other wavelengths such as 700 nm, 800 nm and 900 nm for malic acid and 755 nm for oxalic acid appear to uniquely contribute only to those individual acids. Figure 4 shows the relationship between predicted and actual pH for (a) citric (b) tartaric (c) malic (d) oxalic acids.


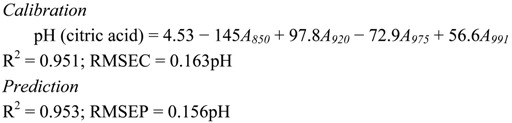
(1)


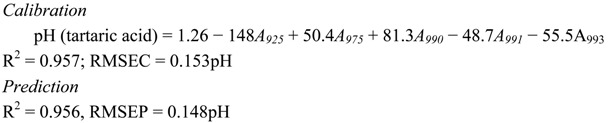
(2)


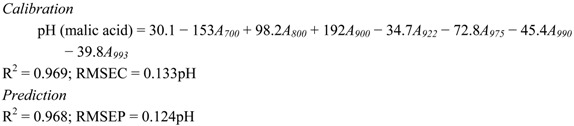
(3)


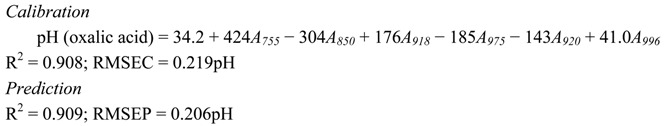
(4)

Table 3 lists the summary of the combinations of important wavelengths in quantifying pH of citric, tartaric, malic and oxalic acids. The squared column in the table indicates the combination of wavelengths that produces the best result for the respective acid.

**Table 3:** Calibration results from MLR using wavelengths from water and pH absorbance bands.

	Acids							
Wavelengths (nm)	Citric		Tartaric		Malic		Oxalic	
	R^2^	RMSE	R^2^	RMSE	R^2^	RMSE	R^2^	RMSE
920, 975, 991	0.914	0.213	0.929	0.193	0.890	0.239	0.319	0.577
850, 975	0.924	0.198	0.902	0.224	0.880	0.248	0.452	0.511
850, 920, 975, 991	0.951	0.163	0.930	0.194	0.909	0.220	0.480	0.509
925, 990	0.909	0.217	0.946	0.166	0.880	0.250	0.217	0.612
900, 975, 990	0.918	0.209	0.925	0.199	0.945	0.170	0.378	0.551
925, 975, 990, 991, 993	0.919	0.212	0.957	0.153	0.887	0.249	0.561	0.473
700, 800, 900, 940, 975	0.927	0.201	0.929	0.197	0.961	0.145	0.673	0.408
700, 800, 900, 975	0.919	0.210	0.926	0.199	0.958	0.150	0.386	0.553
922, 975, 990, 993	0.920	0.209	0.938	0.183	0.883	0.250	0.420	0.538
700, 800, 900, 922, 975, 990, 993	0.928	0.204	0.948	0.172	0.969	0.133	0.465	0.535
755, 850, 975	0.931	0.192	0.907	0.220	0.880	0.250	0.869	0.253
918, 920, 975, 996	0.920	0.208	0.922	0.204	0.897	0.234	0.770	0.339
755, 850, 918, 920, 975, 996	0.945	0.177	0.923	0.208	0.917	0.215	0.908	0.219

## 4. Conclusions

This study has managed to quantify the pH of aqueous acids solutions, *i.e.*, citric, tartaric, malic and oxalic, through the application of NIR spectroscopy between 700 nm and 1,000 nm. The most important water absorbance wavelength for this study is 975 nm. For the pH measurement of all acids solution high measurement accuracy with R^2^ above 0.9 (R^2^ above 0.95 for citric, tartaric and malic acids) have been produced through the wavelengths selected for every acid. Besides, it is sufficient to state the significance of NIR range of wavelengths at 918–925 nm and 990–996 nm in determining acids’ pH since the relatively similar range of wavelengths have been used by other researchers in food acidity measurements.
